# Tendencies Affecting the Growth and Cultivation of Genus Spirulina: An Investigative Review on Current Trends

**DOI:** 10.3390/plants11223063

**Published:** 2022-11-11

**Authors:** Nawal K. Z. AlFadhly, Nawfal Alhelfi, Ammar B. Altemimi, Deepak Kumar Verma, Francesco Cacciola

**Affiliations:** 1 Department of Food Science, College of Agriculture, University of Basrah, Basrah 61004, Iraq; 2 College of Medicine, University of Warith Al-Anbiyaa, Karbala 56001, Iraq; 3 Agricultural and Food Engineering Department, Indian Institute of Technology Kharagpur, Kharagpur 721302, India; 4 Department of Biomedical, Dental, Morphological and Functional Imaging Sciences, University of Messina, 98125 Messina, Italy

**Keywords:** *Spirulina* spp., cultivation, low-cost media, temperature, pH function, biomass, algae toxicity, microbial contamination

## Abstract

Spirulina, a kind of blue-green algae, is one of the Earth’s oldest known forms of life. Spirulina grows best in very alkaline environments, although it may flourish across a wide variety of pH values. There are several techniques for growing *Spirulina* spp., ranging from open systems such as ponds and lakes, which are vulnerable to contamination by animals and extraterrestrial species, to closed systems such as photovoltaic reactors, which are not. Most contaminated toxins come from other toxic algae species that become mixed up during harvest, necessitating the study of spirulina production processes at home. Lighting, temperature, inoculation volume, stirring speed, dissolved particles, pH, water quality, and overall micronutrient richness are only a few of the environmental parameters influencing spirulina production. This review article covers the conditions required for spirulina cultivation, as well as a number of crucial factors that influence its growth and development while it is being grown. In addition, the article discusses harvesting processes, biomass measurement methods, the identification of dangerous algae, and the risk of contaminating algae as it grows on cultures. Spirulina’s rising prospects as food for human consumption are a direct outcome of its prospective health and therapeutic advantages.

## 1. Introduction

Blue-green algae, also known as cyanobacteria, are considered to be one of the first known forms of life on Earth. Algae make up a broad and diversified group of actual nucleus creatures that are known as eukaryotic [1,2]. Its cytoskeleton is a primitive nucleus that has several properties in common with plant nuclei since it is capable of photosynthesis (phototrophic nutrition). The cellular forms of cyanobacteria have developed into a variety of forms, ranging from unicellular forms to multicellular forms. Cyanobacteria can be found in a wide variety of habitats, including aquatic, terrestrial, and marine systems, as well as more extreme or harsh ecosystems such as hot springs, desert soil, certain saline environments, and glaciers [3,4].

More than a thousand years of records show that people have consumed spirulina in the form of food. The blue-green algae spirulina has been a part of human diets for centuries and continues to be eaten in some communities [4]. It was first discovered in Lake Chad in 1940, ten thousand kilometers (km) from Lake Texcoco, another natural source of spirulina. The Kanembu tribe relies heavily on spirulina for protein. They harvest it from the Great Lake (Lake Texcoco) and bake it into a type of bread called “dihe”; spirulina was recognized as “a fantastic food source of the future” by the International Association for Applied Microbiology in 1967. In 1970, Lake Texcoco was the site of the world’s first commercial production of spirulina [5,6]. Between the years 2005 and 2013, the annual commercial output around the globe climbed from 3000 to 5500 tons [7]. *Arthrospira maxima* Setchell and N.L. Gardner and *A. platensis* Gomont are two species of cyanobacteria that are responsible for producing dried biomass that is used in human nutrition. In terms of their place in the hierarchy of taxonomy, these algae are categorized as belonging to the kingdom of bacteria, phylum Cyanobacteria, oscillator Rank, and the Phormidiaceae family [4,8,9,10]. Although spirulina does not produce harmful substances, other species of blue-green algae do. However, the contamination comes from the toxin-producing blue-green algae that are present in spirulina cultures. In addition to this, the level of contamination is higher in the open pond system than it is in the closed bioreactors; hence, the cultivation of spirulina must take place in closed and carefully managed environments. Additionally, in order to prevent the contamination of the produced algae with toxins derived from other algae, the algae must be pure [11,12,13]. Because of this, food regulatory agencies including the European Food Safety Agency (EFSA) and the Food and Drug Administration (FDA) have given their stamp of approval to dietary supplements containing spirulina. A number of environmental factors, such as lighting (light period 12/12, 4 KLux) and temperature (30 °C), inoculation volume, stirring speed, dissolved solids (10–60 g/L), pH (8.5–10.5), water quality, and total micronutrient presence (C, N, P, K, S, Mg, Na, Cl, Ca, Fe, Zn, Cu, Ni, Co, and Se), can all have an impact on the productivity of spirulina [14]. The production of spirulina biomass on a large scale is contingent upon a variety of elements, including the accessibility of nutrients, light, temperature, algal strain, and the mixing and aeration of the culture. Nutrients are the second most important aspect, after cost [15]. The cost of nutrients is one of the primary factors that influence the production of spirulina biomass. Many agricultural media have been produced, employing different types of water, including saltwater, sewage water, and industrial effluents [16]. It is possible to substitute every nutrient in the Xerox Standard media with less expensive and more easily accessible commercial fertilizers and chemicals [17]. Because potassium nitrate (KNO_3_) is typically employed as a nitrogen source, the components of the culture medium are to blame for the high expenses. Additionally, these components have an effect on the behavior of the spirulina. It is possible to replace it with urea, which both slows down and promotes the formation of cells with a larger amount of chlorophyll [18].

This review article explores spirulina, often known as blue-green algae, a kind of cyanobacterium that may be consumed for its nutritional value. Spirulina’s ideal growing circumstances and other aspects that influence its development while being cultivated are also covered in this article. The papers also include how to harvest algae, how to measure biomass, how to identify harmful algae, and how to prevent contamination of algae in cultures. In this article, it is suggested that *Spirulina* spp., which is becoming more likely to be used as food because of its possible health and aesthetic benefits, should be grown safely and scientifically.

## 2. Cyanobacteria and *Spirulina* spp.

### 2.1. Cyanobacteria: Most often Named Blue-Green Algae

Around the year 1300 A.D., the Aztecs began harvesting *Spirulina* spp., also known as *A. platensis* and *A. maxima*, from Lake Ticcoco in Mexico. Spirulina was harvested by the inhabitants of Chad from Lake Kosorum, which is located on the northeasternmost tip of Lake Chad, and consumed on a daily basis for hundreds of years. Furtheremore, *Limnospira fusiformis*, sometimes known as *Arthrospira fusiformis* and commercialized under the brand *Spirulina platensis*, is a spirulina species that is occasionally used interchangeably with *Arthrospira* species. One of the types of cyanobacteria that may be consumed is *Aphanotheca sacrum*, formerly known as *Phylloderma sacrum*. This cyanobacterium is responsible for producing the delectable delicacies known as “suizenji-nori”. In addition to Burma, Thailand, Vietnam, and India, thin green algae such as Spirogyra and Oedogonium are utilized in the food processing industry. Significant advancements in the study of microalgae were initiated in the early forties of the previous century [1,4,19]. Researchers Rippka et al. [20] and Castenholz [21] classified cyanobacteria into five different groups based on the physical distinctions between them, which are as follows: the first section (Chroococcales), the second section (Pleurocapsales), the third section (Oscillatoriales), fourth section (Nostocales), and fifth section (Stigonematales). According to Tomitani et al. [22], these cyanobacteria are unicellular and form threads of varying morphological complexity. Because it does not have a plant cell wall, it has certain traits in common with primitive bacteria. On the other hand, it has some characteristics in common with the animal kingdom due to the presence of complex carbohydrates in the cell membranes, comparable to glycogen. *Nostoc, Spirulina*, and *Aphanizomenon* are all examples of blue-green algae that may be consumed, and their usage as food dates back thousands of years.

### 2.2. Spirulina: An Edible Cyanobacterium with Potential Benefits

Spirulina contributes to the growth of the economy; the alleviation of poverty and malnutrition; and the natural growth of spirulina in alkaline lakes in South Africa, Mexico, Burma, and Latin America, as well as northern Australia and southern Asia. Spirulina is produced using open pond system technology in countries with abundant sunlight and large areas. Spirulina also grows naturally in alkaline lakes using open pond system technology in northern Australia and southern Asia [1,4], and especially in Africa, where species of the genus *Arthrospira* have been collected from the salty and acidic waters of tropical and subtropical regions. *A. platensis* is the most common species within the genus Arthrospira, and it is found mostly in Africa and Asia. On the other hand, the *A. maxima* have been discovered in both California and Mexico [8,23]. *Spirulina* spp. reproduces via fragmentation and elongation as a result of the self-destruction of the algal thalli into fragments. These fragments then develop and expand in length by binary fission while concurrently taking on a spiral form [5,15,24]. *A. platensis* requires certain environmental parameters in order to thrive, including an aquatic environment with a pH between 9.5 and 10.5, a high concentration of sodium carbonate salts at a rate of 10–20 g/L, and a temperature that ranges between 35 and 37 °C throughout the day. These circumstances allow for the fast growth of these algae while suppressing the development of other algae, such as those belonging to the genus *Chlorella* [18,25,26,27]. 

**Figure 1:**
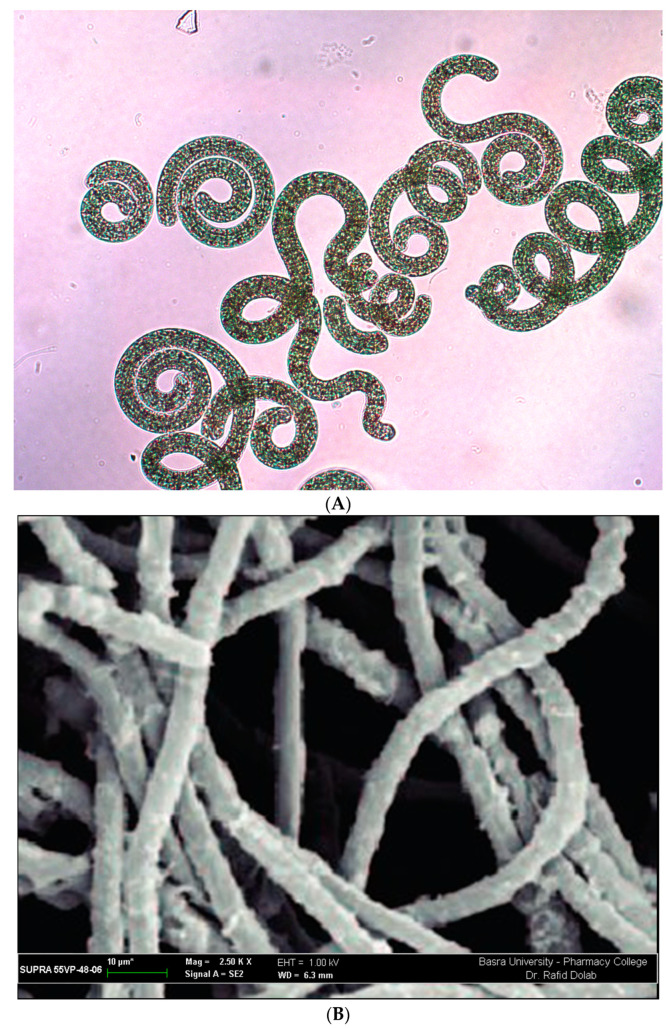
Instrument’s perspective on the shape and structure of *Arthrospira platensis*. (**A**) Microscopic view and (**B**) scanning electron microscopic view.

According to *Bergey’s Manual for Bacteriology*, which explains that *Spirulina platensis* and *A. platensis* differ in shapes such as snail type, cell wall, and the microscopic view of diameters and filaments, *S. platensis* was first isolated from Lake Texcoco by the Aztecs in the 16th century. *S. platensis* is found among eukaryotes [1,4,28]. According to the study of botany, the name *S. platensis* was formerly referred to as *A. platensis* because of the oxygenic photosynthetic feature of the plant. Spirulina is the name that scientists from all around the world are currently using to refer to various types of microalgae [1,4,21,28,29,30]. The genus *Spirulina* includes 15 distinct species. The term originates from the distinctive spiral or spiral form of filaments, also known as trichomes, which may be seen in all different kinds of cyanobacteria [1,4]. According to a statement made by the Food and Agriculture Organization (FAO) in 2008, species of *Arthrospira* that belong to the genus *Spirulina* are referred to as spirulina since members of this genus have been studied and utilized the most [14,23]. The shape and structure of *Spirulina* spp., as shown under a microscope, are explained (Figure 1A) [21]. However, *Spirulina* was shown to belong to the genus *Arthrospira* based on its morphological appearance under a scanning electron microscope, as shown in Figure 1B, as well as by employing the genetic sequence analysis of 16S rRNA [31,32].

This procedure was more accurate in the past and still is more accurate now when it comes to establishing the identity of microorganisms. The classification of *Spirulina*, also known as *A. platensis*, can be seen in Table 1. The product that is more often known as *Spirulina* is really a member of the genus *Arthrospira*. Owing to the long use and marketing of many products such as *Spirulina*, which is generally used for the two most significant products as food (*A. platensis* and *A. maxima*), these words are used interchangeably. The reason for this is due to the long use and marketing of many products as spirulina [21].

## 3. Toxicity of Blue-Green Algae

Toxic chemicals called aquatic algal toxins represent a diverse range of poisonous substances that are naturally generated by microalgae and blue-green algae. These substances are harmful to human health and pose a threat to the environment. Secondary metabolites, also known as cyanotoxins, are generated by cyanobacteria and are recognized to be hazardous to both human health and the environment. Blue poisons are also known as cyanotoxins. Drinking water and dietary supplements containing algae are the sources of these poisons. The afflicted parts of the body determine the chemical makeup and level of toxicity of these poisonous chemicals, which might vary. Microcystins are the most frequent form of blue poison. Abdominal discomfort, vomiting, diarrhea, skin irritation, weakness, and a sore throat are some of the symptoms of cyanotoxicosis that can occur after consuming these products [12,13,31]. These symptoms can be caused by cyanotoxins. Blue-green algae naturally produce three different kinds of cyanotoxins: a-anatoxin and methylamino-L-alanine, microcystin-LR, and cylindrospermopsin (CYL). The World Health Organization (WHO) has advised a limit of 1 µg/L of MC-LR in drinking water [12,33,34]. Because the majority of toxins that contaminate spirulina are sourced from other forms of harmful algae that are combined with them during harvest, Tang and Suter [35] noted how important it is to investigate home cultivation practices for spirulina. In their research, Murugesan et al. [36] highlighted the potential that the cells of *Spirulina* spp. might play a role in the removal of heavy metals from aqueous solutions through the absorption of bio-cadmium by live cells. The highest amount of absorption that could be obtained was 44.56 mg/g. As a result, spirulina is an excellent candidate for use as a biosorbent material. This was demonstrated by the fact that the amounts of heavy metals in commercial spirulina products were found to be within the acceptable range. The production of spirulina calls for the utilization of high-quality nutrients and the precise determination of heavy metals in the cultivation environment as well as in the biomass. Additionally, a great deal of caution should be exercised during the cultivation of spirulina in order to avoid the contamination of the crop with heavy metals or other cyanobacteria that are capable of producing toxins [8,10]. When analyzing the main toxicity of tested cells with human red blood cells, Nair and Thompkinson [37] revealed a method that is now used as one of the usual ways for identifying algal toxins. Nowadays, this approach is used in the study. An indication of the toxicity of substances that were tested is provided by the concentrations that were measured using erythrocytes. This is because of the formation of a covalent bond between the toxic compounds and the free radical of the glutathione protein, and the rate of degradation is determined by the concentration of the substance, the length of time that the incubation process is allowed to continue, and the temperature.

**Figure 2:**
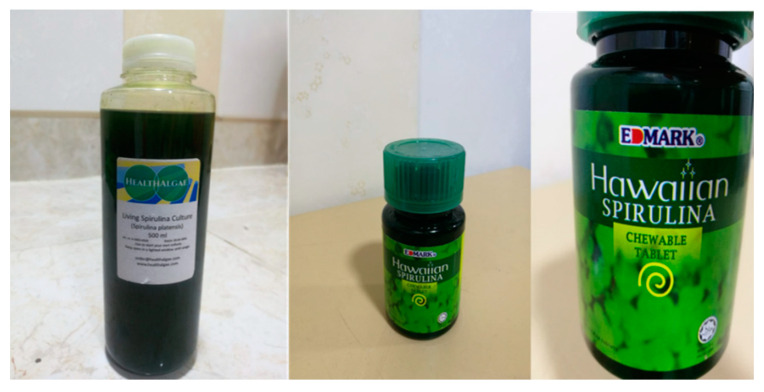
Various Spirulina-based products developed for commercialization.

Spirulina supplements have piqued the interest of a significant number of researchers, who have discovered that they are contaminated with blue toxins. Low quantities of blue toxins were found in the 31 spirulina products that were tested for toxicity. These toxins do not provide any health hazards to adult consumption, but they do pose health problems to children and babies when ingested on a daily basis [13]. In addition, 18 algal food supplement products were tested for the presence of toxins, and the results showed that 8 of the products contained cyanotoxins at concentrations that were higher than the threshold considered safe for daily use. According to the research of Roy-Lachapelle et al. [11], consuming 10–19 g of spirulina per day, and even up to 40 g in Africa, over an extended period of time did not result in any adverse effects for consumers. Spirulina supplements have been deemed “potentially safe” by the National Institutes of Health in the United States, providing that they are free of microcystin contamination [12]. Figure 2 presents a variety of blue-green algae in its many forms. 

In their research, Roy-Lachapelle et al. [11] also demonstrated that out of the 14 different kinds of spirulina dietary supplements, 3 of them had microcystins. When rats were given a diet containing 5% phycocyanin from spirulina for 14 weeks, the rats had no toxicity symptoms, making them safe for human consumption [38]. The study explained that spirulina typically cannot produce blue toxins at a rate ranging between 0.25 and 0.84 µg/g of algae based on the dry weight. This information was gleaned from the results of a study that examined the ability of spirulina. In addition, Bashir et al. [39] evaluated the safety of *Spirulina* spp. by giving rats diets that were augmented with *Spirulina* spp. and pure spirulina protein for a period of 45 days. This was carried out in order to perform the study. It was revealed that it does not contain any toxins, which means that it does not have any adverse effects on the spleen, liver, or kidneys. According to the findings of Hutadilok-Towatana et al. [40], administering a methanolic extract of spirulina to rats at dosages of 6, 12, and 24 mg/kg body weight on a daily basis for a period of 12 weeks did not produce any signs of toxicity in the experimental animals. *Spirulina* spp. were put through a cytotoxicity test by Liu and Cao [41] using a normal cell line of human prostate myofibroblasts (WPMY-1) as the test subject. The concentration of the methanolic extract of spirulina ranged from 250 to 1000 µg/mL, and the researchers came to the conclusion that the extract was safe for human consumption. Additionally, they stated the health-preventive role of *S. maxima* on feeding rats at a dose ranging from 0.5 to 1.0 g/kg of body weight, and they found that eating 1.5–4.5 g of body weight for 90 days had a positive effect on the animal’s health. An aqueous extract of *Spirulina* was given a cytotoxicity test at a concentration of 300 mg/mL [42], and the study came to the conclusion that the extract did not cause any cytotoxicity to human red blood cells. As a result, it is both risk-free and non-hazardous, making it suitable for usage in the pharmaceutical sector.

## 4. Growth Conditions and Factors Affecting Cultivation of *Spirulina*

There are a variety of methods for producing *Spirulina* spp., but the two primary technologies are open systems such as ponds and lakes, and closed photovoltaic bioreactors. Because they are closed systems, photobioreactors in the environment do not permit the direct interchange of gases and contaminants with the surrounding environment. They make it easier to regulate aspects of the cultivation environment such as the supply of carbon dioxide and water, the ideal temperature, the intensity of the lighting and culturing, the pH, and the gas exchange and ventilation [42]. By including carbon dioxide in the media, spirulina cells were grown and then moved to outdoor raceway tanks where they were maintained at 33 °C, 7000 Lux, and pH 9.0–10.0. The spirulina cells were dried at a temperature of 60 °C for one hour, and the dried samples were then subjected to a rigorous crushing process before the algae powder was compressed into tablets [1]. Spirulina that is grown in closed environments yields biomass that is free of contaminants and contamination, which makes the process of its production and incorporation into food more straightforward [18]. 

Nutrients not only impact the metabolism of microalgae, but also their development rate and the composition of the finished product [43]. The most abundant form of carbon (C) may be found in culture media and inorganic minerals including nitrates and micronutrients (such as F, Mg, and Mn). The compounds nitrate (NO^3–^) and nitrite (NO^2−^), as well as ammonia (NH^4+^) or urea, are utilized to provide the element nitrogen (N_2_). They are necessary for the production of amino acids, which are building blocks for proteins, as well as the expansion of microalgae, and these nutrients have to be accessible in a well-balanced form [15]. Growth variables such as light intensity, temperature, pH, and salinity can have a major influence on the lipid and pigment content in cells of spirulina, which determines the composition of nutrients in spirulina biomass and is dependent on these growing parameters [1,44]. 

The growth of *Spirulina* spp. can be stimulated by raising the concentration of ammonium nitrate, which results in a maximum biomass of 0.353 g/L. On the other hand, the growth slows down when the concentration of urea is raised. It was observed that the standard medium had less biomass, chlorophyll, and protein than the experimental medium did (0.840 mg/L, 0.0701 g/L, and 52.95%, respectively). The highest biomass, chlorophyll, and protein were detected in the experimental medium [17]. 

### 4.1. Growth Medium Employed for Spirulina Cultivation

At a pH range of 8.0–8.3, the biomass of *Spirulina* spp. that was grown in saltwater with urea was 7.35 g (dry weight)/m^2^/day. This was a lower value than the biomass of *Spirulina* spp. that was grown in seawater with sodium bicarbonate (NaHCO_3_), which was 8.14 g/m^2^/day [14,44]. The impact of nutrients on the amount of biomass produced by spirulina was studied by observing how the addition of various nutrients (carbon in the form of sodium bicarbonate, nitrogen in the form of urea, phosphates, sulfates, iron, magnesium, and potassium) affected the rate at which algae grew. It was determined to be 0.78 g/L (based on dry weight), and after 40 h of cultivation, the growth rate increased to 0.82 g/L after sodium bicarbonate was added. At a temperature of 30 °C, it was observed that using urea as a source of nitrogen in spirulina made its absorption by the spirulina easier, which resulted in an increase in biomass, but had no effect on the chlorophyll content [14,44]. A vibrating culture medium supports the growth of *Spirulina* spp. in a laboratory environment.

It was demonstrated that the choice of the culture medium and the length of its period are determined by the requirement for the ultimate product, which may be biomass or pigments. Spirulina was grown in four different cultivation media in order to find the optimal medium for producing biomass and pigments under the following conditions: a development temperature of 30 ± 2 °C; a light intensity of 4.5 KLux m^2^ mediated by fluorescent lamps; and an inocula volume of 10% (volume/volume) of the total volume of the cultivation medium. The cultivation duration of 30 days was the optimal period for the generation of maximum biomass, which was 4.87 g/L for the standard medium Xarox. This maximum biomass production was achieved during the optimal cultivation period. This is because of the high alkalinity of the substance (pH 8.2). At 20–30 days of cultivation, the modified BG-11 medium acquired the highest production of chlorophyll, carotenoids, phycocyanin, and allophycocyanin; however, the medium SHU achieved the maximum production of phycoerythrin [45]. 

There are great deals of different nutrients that are required for the growth of algae. In phytoplankton cultures, selecting the source of the nitrogen and the concentration of the nitrogen contributes to the modification of metabolic activities and, as a consequence, the components and nutritional value of the microalgae. This is why selecting the source of nitrogen and the nitrogen concentration is so important. Phosphorous is an essential component that is essential to playing a major role in sustaining high rates of production of microalgae. As K_2_SO_4_ was substituted by utilizing commercial fertilizers, commercial agriculture fertilizers can be considered a source of phosphorous. Murat potassium MOP is a source of potassium, and raw sea salt is a source of micronutrients that are required for the growth of spirulina [17].

The key component that controls the growth and production of spirulina is the nutritional circumstances that it is exposed to. Potassium is one of the nutrients that spirulina needs in order to grow, and the intense growth of the algae may be controlled by switching from potassium nitrate to sodium nitrate and altering the source of the growing media. Growth and biomass were both improved when the Xarox Standard Medium was substituted with other commercial ingredients, such as single superphosphate (SSP), instead of dipotassium hydrogen phosphate, EDTA, and micronutrients A5 (such as boric acid, manganese chloride, zinc sulfate, sodium molybdate, and copper sulfate). Potassium is an essential component that plays an important part in the provision of a suitable ionic environment for metabolic processes in the cytosol. Potassium is also a regulator of various processes, such as growth regulation, photosynthesis, and protein synthesis, and it is a source of nitrates in the medium, which increases the concentration of biomass. Because of its high cost, micronutrient A5 is not utilized in the process of identifying the growth of the strains. On the other hand, the modified Xarox medium, which does not contain any micronutrient A5, combined with the addition of potassium nitrate and sodium nitrate, resulted in the optimal growth of spirulina with regard to a specific growth rate, biomass, and chlorophyll A [16].

In batches of cultivation spanning for 28 days, it was revealed that the modified Xarox medium might be diluted up to five times without having an effect on the growth rates. After 21 days of batch cultivation (1.21 g/L), higher dry weights were reported for 20% of the regular Zarrouk medium compared to 0.84 g/L for the Zarrouk-modified medium. It was found that the nitrogen content of 2.5 g/L was ideal for the medium, and that urea was a superior source of nitrogen to either ammonium or nitrate as a fertilizer ingredient. It was also reported that it had a substantial influence on the productivity of spirulina and that increasing the phosphate concentration to 250 mg/L in the form of potassium phosphate K_2_HPO_4_ boosted the production of biomass [44]. The exocytosis of spirulina could be maintained in its purest form at a bicarbonate concentration of 0.2 M, which was the lowest concentration tested [24].

Since carbonate is not used in the cultivation process, urea and bicarbonate are the primary components of the growing media. If fertilizer-grade chemicals rather than low-cost substitutes are employed, the potassium content can be raised; however, this must be completed with caution so that it does not go over five times the concentration of sodium. It should not be difficult to dissolve or granulate; rather, it should be crystalline or soluble [40]. In order to carry out photosynthesis, the medium in which the algae is grown must have carbon. Because spirulina contains 47% of the carbon that is required for its growth, the medium in which it is grown has a very high concentration of sodium bicarbonate (10.8 g/L) and sodium carbonate (7.6 g/L) [44]. Numerous minerals, including potassium phosphate, potassium sulfate, sodium (sodium carbonate, sodium bicarbonate, sodium nitrate, and sodium chloride), phosphorous (potassium phosphate), calcium (calcium chloride), magnesium (hydrated magnesium sulfate), iron (ferrous sulfate hydrate), and EDTA, are required for the growth of spirulina and must be present in the culture medium [44]. Despite the fact that the spirulina medium has modest concentrations of iron (2 mg/L), it has a rather significant quantity of iron (0.58–1.8 g/kg) in its biomass [44]. 

### 4.2. Low-Cost Media for Spirulina Cultivation

Spirulina may be cultivated in many different types of industrial and agricultural waste, including waste from sugar mills, trash from the poultry industry, waste from fertilizer factories, waste from digested fowl, ash from banana leaves, garbage from municipalities, and organic materials. The most significant obstacles to growing spirulina are the associated costs and the limited availability of inorganic minerals. In the cultivation of spirulina, there is an option to employ organic sources of nutrients, particularly liquid waste from the fertilizer industry. This alternative results in yields of 11% (*w*/*w* dry matter) and contains phosphates, nitrates, and sulfates with a pH ranging from 7.4 to 8.5. One of the wastes that are utilized as a source of carbon is wastewater from Thai fermented noodle manufacturers. This wastewater, when combined with filtered seawater with a pH of 8.30, may be a source of nutrients for the algae spirulina [14]. When growing spirulina, rice husk ash and sodium bicarbonate were employed as carbon sources. However, adding 2 g of sodium bicarbonate per liter of water every two days resulted in higher growth of the spirulina compared to adding 1 g of rice husk ash per liter of water every day [1]. Mono superphosphate, sodium nitrate, potassium murate, sodium chloride, magnesium sulfate, calcium chloride, and bicarbonate were the ingredients used to produce a low alternative medium for the cultivation of spirulina (RM6). The growth rate was compared with Zarrouk’s standard medium in terms of dry biomass, chlorophyll, and proteins, and the results were very close to the two media. However, the alternative medium was less expensive at USD 16 per liter in comparison to the cost of the standard medium, which was USD 79.5 per liter [14].

Spirulina was cultivated by Lemes et al. [46] by exchanging potassium nitrate (KNO_3_) for urea as a nitrogen source. The added amount of urea was 2.5 g/L, and it was spread out over the course of 14 days at a temperature of 30 °C. An inoculum was used at a concentration of 50 mg cell/L, and the stirring speed was 180 rpm. The light intensity was 42 μmoL. The growth of photons/m^2^/s over a period of seven days was determined by measuring the optical density, and it was discovered that the replacement was helpful in increasing the proportion of protein while having no effect on the synthesis of chlorophyll, which resulted in a reduction in the cost of cultivation. The purpose of the cultivation of *Spirulina* spp. in wastewater is to produce biomass while also removing organic and inorganic pollutants, providing an alternative to traditional purification treatments, enhancing the activity of photosynthesis, and increasing the accumulation of phycocyanin and chlorophyll type II. It has been noticed that heavy metals and other foreign substances are removed from water bodies as a result of algae growth on effluents [1]. The digested effluent is a source of low-cost nutrients C:N:P in a ratio of 24:0.14:1, which supports the growth of spirulina. This increased the proportion of protein, carbohydrates, and fats in biomass to 68, 23, and 11%, respectively, while the percentage of chemical oxygen demand (COD) and levels of ammonia, nitrogen, and phosphate in wastewater decreased to levels of 98.0, 99.9, and 99.4%, respectively [1]. Spirulina was also grown on a low-cost medium of latex serum containing mineral salts and organic compounds. This Mg:P:N:C medium in a ratio of 0.2:0.3:3:1 gave 0.350 g/L of carotenoids of biomass, whereas the industrial medium was 0.407 g/L for two months [1].

This low-cost medium has been prepared by replacing all of the nutrients in the Xerox standard medium with cheaper and more locally available commercial chemicals and fertilizers. These cheaper commercial chemicals and fertilizers are mono superphosphate, commercial sodium bicarbonate, muriatic potash, and raw sea salt. This has allowed the medium to be prepared as a low-cost medium. Ammonium nitrate or urea, two other sources of nitrogen, were chosen to take the place of sodium nitrate as the primary source of nitrogen. It was revealed that the type of nitrogen source and concentration had an effect on the levels of protein, carbohydrates, and fats produced by spirulina. Additionally, it was found that the prepared medium, with ammonium nitrate added as the nitrogen source, grew spirulina in a manner that was comparable to that of the standard medium in terms of dry biomass, chlorophyll, and chemical composition. It was determined that the cost of a liter of the normal medium was USD 80, whereas the cost of a liter of the ammonium nitrate medium was just USD 13 [17]. The effect of the low-cost modified standard medium on the growth of six different strains of spirulina was investigated in the study of Rajasekaran et al. [16]. The culture temperature was set at 30 °C and cold-white fluorescent bulbs were used as the source of lighting (2500 Lux). The rate of rotation of the culture was 75 rpm, and the photoperiod of light and dark cycles was 12/12. The inocula volume was 10% (volume/volume). Over the course of 40 days, measurements of dry weight were taken every 5 days, and maximum biomass of 5 g/L was determined [16].

The fact that spirulina media have nutritional contents comparable to those of Zarrouk’s medium has an impact on the prices of these media. It is possible for inexpensive media to produce satisfactory levels of biomass, chlorophyll, and protein content. Zarrouk’s medium resulted in superior development compared to the other media that were evaluated; nevertheless, it included a greater quantity of chemicals, which led to a rise in the price of those chemicals. It had a maximum biomass concentration of 4.6 g/L, which was due to the nitrogen content that it contained. The selection of the source and concentration of nitrogen by Delrue et al. [47] is significant in the cultivation of phytoplankton because nitrogen influences the metabolic activity, components, and nutritional value of algae. In the field of aquaculture, this is an essential factor to take into consideration [17]. When added to large outdoor ponds, certain strains of spirulina have shown that they are able to use alternative nitrogen sources such as low-cost ammonium and urea rather than high-cost nitrate and phosphate. These strains have also demonstrated the activity of the phosphatase enzyme, which provides the phosphate element even in environments where it is scarce [48]. Michael et al. [49] investigated the effect of the medium type on the antioxidant activity of spirulina *A. fusiformis*, cultivated in a low-cost medium (LCMA), and compared it with Zarrouk’s standard medium. They reported that the LCMA medium was superior to the standard medium due to the fact that it contains a higher quantity of natural antioxidants as a result of the use of a low-cost inorganic cultivation medium. This finding was published in the journal “*African Journal of Food Science*” in 2018. Ranjith et al. [50] determined the growth rate of spirulina in a modified NRC medium by exchanging urea and phosphoric acid for sodium nitrate and potassium dihydrogen phosphate (anhydrous). Additionally, the concentration of ferrous sulfate was decreased, and the spirulina was grown in an indoor cultivation setting with the illumination of 3500 Lux, a light/dark cycle of 12:12 h, and a temperature of 24 °C. When compared to the modified NRC medium, Zarrouk’s standard medium had a growth rate value that was 5.7% higher, but the modified NRC medium’s cost was much cheaper than that of the standard medium [50].

A low-cost medium for the cultivation of *S. platensis* was developed by exchanging the nutrients included in Zarrouk’s medium for urea fertilizer and soil extract that contained varying quantities of carbon sources [51]. The highest rates of growth and production were 0.50/day and 1.05 g/L in the modified medium (7.50 g/L of sodium bicarbonate and 2 g/L of urea without sodium bicarbonate) compared to the rest of the media, while protein (56.14%), carbohydrates (16.21%), and a high concentration of glutamic acid (14.93%) were produced. Ten media with different concentrations of nitrogen were prepared from urea and sodium bicarbonate [51]. The findings of the research led the scientists to the conclusion that spirulina may be grown at a cheap cost using a low concentration of urea, sodium bicarbonate, and soil extracts.

Researchers analyzed the biomass, chemical composition, and beneficial compounds of spirulina *A. fusiformis* grown for 28 days in glass aquariums under indoor conditions in standard Zarrouk’s medium and a low-cost medium (LCMA) containing N:P:K (10:20:20) commercial fertilizer as a source of the three major nutrients essential for spirulina growth [49]. Because of its high chlorophyll content (0.9%) and dry weight (0.75 g/100 mL), the LCMA medium promoted the healthiest and most robust development. When compared to Zarrouk’s medium, the protein content was greater than 50 % of the dry weight, and therefore believed that it was a suitable medium for enhancing vitamins and certain minerals, including salt and potassium [49]. The production of spirulina biomass can also make use of low-cost media such as seawater and sewage water from shrimp hatcheries. The researchers found that the growth rate was reduced by 3–14% in the modified seawater medium in comparison to the standard medium. This was determined by analyzing the protein and pigment contents of the seawater. The pigments discussed in the modified NRC medium were phycocyanin, chlorophyll A, and total carotenoids. The concentration of phycocyanin in the modified saltwater was 50.9 mg/g, while the concentration in the normal medium was 50.95 mg/g [52]. 

### 4.3. Temperature

**Figure 3:**
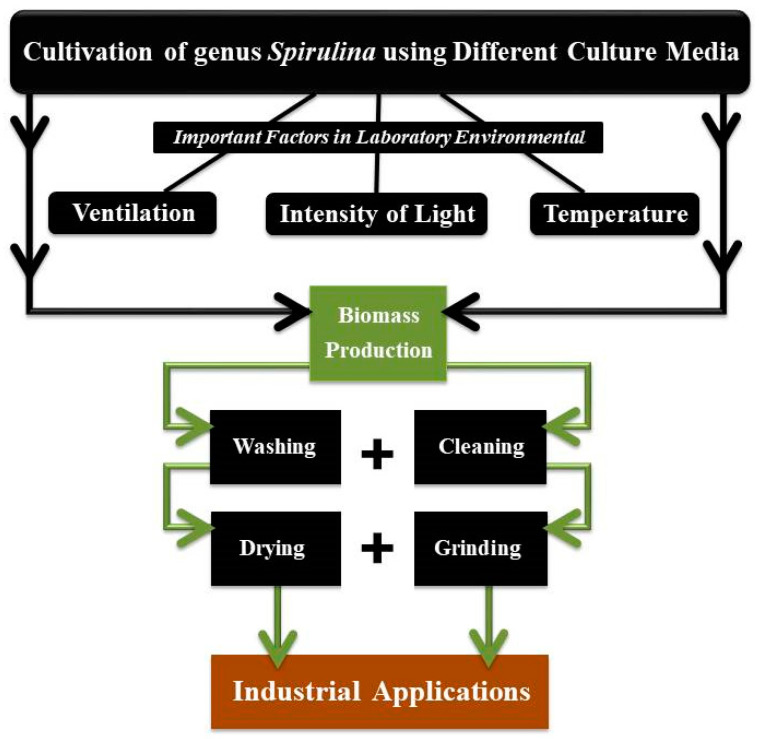
Key procedures for the growing and production of spirulina under laboratory conditions.

When grown in a laboratory, the ideal temperature for spirulina cultivation ranges between 35 and 37 °C. However, when grown outdoors, the temperature can rise to 39 °C for a few hours without affecting the ability of blue-green algae to photosynthesize. Temperature plays an important role in the cultivation of spirulina. The optimal temperature for spirulina cultivation is between 35 and 37 °C. Spirulina can be cultivated at temperatures between 35 and 40 °C, which allows the thermophilic microbial contaminants of moderate severity to be eliminated. Spirulina strains that thrive in heat as well as those that are resistant to heat can both be grown at these temperatures. It has been shown to produce the phenomena of nocturnal loss of vital mass, as well as increase the respiratory rate during the night. The lowest temperature at which spirulina may be grown successfully is around 15 °C throughout the day [14]. A flowchart of the processes involved in the cultivation and production of spirulina in the laboratory is presented in Figure 3.

It was revealed that the loss in biomass that occurs as a result of night-time respiration is greater in algae that are cultivated at a temperature of 35 °C [14]. At 25 °C in the morning and evening, the photosynthetic activity of the culture shifted from being directed towards the synthesis of carbohydrates from it towards the synthesis of protein. It was also noted that the amount of β-carotene was reduced. At a temperature of 25 °C in the morning and evening, the flow of valence electrons was aimed in the direction of the synthesis of protein [14]. The outside temperature of 38 °C was the most ideal for producing the maximum yield of spirulina, and any temperature over 35 °C leads to bleaching of the culture. Temperatures outdoors can range from 0 to 50 °C. Usharani et al. [53] investigated the effect that a temperature range of 25 to 34.5 °C had on the cultivation of spirulina *S. platensis* under a light intensity that ranged from 15 to 69 μL photons/m^2^/s, while the nitrogen source was either potassium nitrate or urea. Using urea and estimating the cell concentration resulted in a cell yield of around 95 mg/L. The optimal temperature for growth was determined to be 30 °C, and the optimal light intensity for growth was 60 μL photons/m^2^/s. According to Danesi et al. [18], the influence of temperature on the development of spirulina was evident throughout the winter since the daily temperature declined, eventually reaching around 10–15 °C in the middle of the winter. The pond was covered with a polyethylene sheet that was 0.2 mm-thick, which resulted in a temperature rise in the culture medium that was 5–7 °C higher than the temperature of the pond while it was open. This temperature increase had an effect on the culture [54]. Thus, the temperature is one of the most important environmental factors that can influence the rate at which spirulina grows. Spirulina does not grow below 17 °C, but it does not perish above 38 °C. The optimal temperature for growth is 35 °C, but temperatures that are higher than 38 °C either prevent or disrupt its growth [1,44]. It was demonstrated that temperatures of 35 °C reduced the amount of biomass produced by spirulina, while temperatures of 30 °C promoted its growth. This is because spirulina is able to consume carbon dioxide (CO_2_) quickly (ten to fifty times faster than terrestrial plants due to its rapid growth rate) and convert it into biomass through the process of photosynthesis (Mogale, 2016). The quantity of biomass, the metabolic processes, and the production of spirulina cells were all found to be affected by temperature, as revealed by the researchers. The maximum growth of spirulina was reached with an increase in chlorophyll from a temperature of 25 °C, while the highest concentration of biomass was attained at 30 °C. The temperature of 35 °C offered the maximum growth of spirulina. The loss of biomass that occurs throughout the night is dependent on the reduction in temperature, cell density, and light radiation. Temperature is one of the factors that play a role in this loss. The reduction in temperature causes a rise in the degree of unsaturation of the fatty acids that are present in the membrane lipids. These fatty acids are responsible for the maintenance of membrane fluidity, which is necessary for the proper functioning of biological membranes [47,48,55]. 

### 4.4. Water Quality

The characteristics of the water quality contributed to the mass production of algae and had a double effect by affecting the solubility of added nutrients in the agricultural media and the accumulation of some heavy metals by the algae while they were in the growth stage. In addition, the water quality characteristics had an effect on the mass production of algae [44,45,46]. Spirulina can be cultivated in open ponds and natural waterways such as lakes, ponds, reservoirs, and even constructed ponds and reservoirs. Ponds that are shallow and huge, as well as circular ponds, reservoirs, and raceway ponds, are the types of systems that are utilized the most frequently. Open systems are the most straightforward to construct and run, which leads to reduced costs of both production and operation [1,41,42]. The water that is used to cultivate spirulina has to be clean and filtered to prevent the growth of other algae. Although the water often includes an adequate amount of calcium, it promotes sludge production if it is too difficult. Spirulina can be grown successfully with both reverse osmosis (RO) and portable water; however, portable water is preferable [1,41,42].

### 4.5. Salinity

Spirulina can be found in soil, swamps, freshwater, brackish water, seawater, thermal springs, and alkaline salt water (>30 g/L) with a high pH (8.5–11.0) [23,45,55]. Its optimal growth is with a salt ratio ranging between 20 and 70 g/L and high sunlight in the tropics, which helps with the good production of *Spirulina* spp. [14]. Under ideal conditions, the cellular osmotic composition of spirulina is balanced with the environment. However, under unbalanced conditions, cells suffer from osmotic stress, which leads to physiological changes that either increase or decrease the production of biochemical compounds and affect the integrity of spirulina cells. In addition, the osmotic composition of spirulina cells is balanced with the environment under ideal conditions.

Osmotic stress may be caused by either a lack of salts in the culture medium or an excess of salts in the cultivation medium. The excess salts enhance the salinity rate and accelerate the production of carbohydrates while limiting the synthesis of fat and protein [15]. Due to the high nutrient and salt requirements of spirulina, it can only be naturally cultivated in salt lakes [44,45,46]. Spirulina has the ability to grow and adapt in environments with high salt, and it can be cultivated in salinities ranging from 10 to 40 psi without experiencing substantial changes in its biomass dry weight, chemical composition, or fatty acid levels [44,45,53]. 

### 4.6. Light Intensity

Through a process known as photosynthesis, all phototrophic organisms, including higher plants, photosynthetic bacteria, and cyanobacteria, are able to transform the energy from light into chemical energy. High light intensity affects the maximum growth rate, while a low light intensity leads to the synthesis of biomass rich in pigments and proteins, increases the concentration of cultured cells, increases self-shading, and leads to a decrease in the growth rate of spirulina. Both the quality and intensity of light are important factors in the production of algae [1,42]. The majority of photosynthetic plant cells, including those of spirulina, derive most of their required energy from light. They are capable of capturing light energy from the sun or from artificial light sources, and they convert carbon dioxide into oxygen while doing so. Their growth is slowed when the amount of light available to them is lowered, and in photobioreactors that are lighted from the outside, the cells closest to the surface of the reactor receive the majority of the light, while those in the center receive less light. When exposed to extremely high light intensities, the cells eventually become saturated and are unable to utilize the additional light; as a result, the phenomenon of photodamage takes place. *S. platensis* and *S. maxima* are well-known species that grow at a high optical density that eventually reaches saturation. Their light intensities range from 150 to 200 μmoL photons/m^2^/s and from 420 to 504 μmoL photons/m^2^/s, respectively. Both of these densities are considered to be saturated [15]. When selecting a light source, it is essential to make sure that the spectral characteristics of the light flux and the wavelength range are appropriate for photosynthetic organisms. These organisms may have specific requirements for the amount of light, how long they must be exposed to it, and what wavelength range they must fall within [55]. When the culture is moved, the optimal light intensity for spirulina cultivation and biomass production varies from 250 to 350 W/m^2^, and the maximum light intensity is 400 W/m^2^, which is the point at which the growth of the culture ends [47]. Because the temperatures in the culture were high enough so that light is the primary factor that controls the growth of spirulina in the external environment, the increase in the concentration of cells in the culture led to an increase in self-shading and thus a decrease in the growth rate during the summer. On the other hand, the effect of self-shading was less obvious during the winter and spring because the temperatures on the outdoor cultures were lower, so the effect of self-shading was less noticeable during those seasons [14]. An increase in the light intensity up to Lux led to a rise in the content of β-carotene, and the growth of spirulina cells under the red color gave the highest content of β-carotene, followed by blue light and white light, respectively [14]. It has also been reported that the shade of spirulina cells might diminish the photosynthetic activity of those cells. This indicates that these cells require a higher quantity of light in order to achieve the same level of activity as non-photosynthetic cells. During the growth phase, spirulina needs an optimal light intensity between 20 and 30 KLux, and the optimal photoperiod is 16 h per day, according to an analysis of the chlorophyll content of the plant and the light intensity [14,53]. 

Usharani et al. [53] investigated how the intensity of light affected the development of spirulina. It was highlighted that the dry weight of spirulina was 0.85 g/500 mL at a light intensity of 5 KLux, and the contents of protein and chlorophyll A were 64.3% and 9.8 mg/g, respectively. This was in accordance with the findings that the light intensity during the cultivation of spirulina should be moderate in order to prevent the photolysis of the cells. According to Danesi et al. [18], this intensity of 60 µmol photons/m^2^/s is sufficient to obtain the maximum growth of the cells. When the light intensity was increased to higher values, there was no increase in cell growth due to the shadow effect, which is responsible for the stunting of growth. According to the specific growth rate at a light density ranging from 85 to 430 μmol/m^2^/s, Delrue et al. [47] provided an explanation of the influence that the light intensity had on the spirulina biomass production in a photobioreactor with a capacity of 2 L. They did not see any photoinhibition even though the light intensity was 430 μmol/m^2^/s, and the greatest growth rate was 0.12 per day. Because spirulina can only grow in the presence of light, lighting is an essential component of spirulina culturing. However, lighting the spirulina 24 h a day is not ideal because many chemical reactions, including protein synthesis and respiration, take place inside the spirulina during the dark periods of the day [1]. 

### 4.7. pH

For spirulina to flourish, the environment in which it is grown must be quite alkaline, and it must have access to bicarbonate ions. The rise in carbon dioxide levels in the atmosphere has an influence on photosynthesis as well as the development of spirulina; nevertheless, it did not produce any change in the maximum growth rate, a loss in biomass productivity, or any effect whatsoever on the number of pigments in algae [47]. The acidity function not only determines the solubility of the carbon and mineral sources in the culture but also directly influences the physiological properties of algae and the availability of nutrients. Indirectly, the acidity function affects the degree to which carbon and mineral sources are soluble. Spirulina thrives in environments with a pH ranging from 9 to 11, and its pH rises from 8.4 to 9.5 throughout the cultivation process as a result of the consumption of bicarbonate and sodium ions. At a pH of 9, the dry weight was 0.91 g/500 mL, and the percentages of protein and chlorophyll A were 64.3% and 13.2 mg/g, respectively. In addition, the acidity function had an effect on the development of spirulina as well as protein and chlorophyll content [47]. It was shown that the optimal growth of *S. platensis* occurred at a pH level of 10, which may be related to the fact that the activity of all enzymes necessary for photosynthesis and respiration is maximized at this pH level [53]. When analyzing the parameters that influence the amount of beta-carotene, Ahsan and colleagues found that the rate of cell proliferation slowed down significantly between pH 10.5 and 11.0 [14]. The effects of variations in pH (ranging from 7.5 to 11) on antioxidant activity and spirulina production were investigated by Madkour et al. [17]. At a pH of 9, the algae had the highest dry weight, the maximum protein production, and the highest antioxidant activity. However, at a pH of 8.5, the algae had the highest content of chlorophyll and carotenoids, and at a pH of 9.5, the algae had the highest phenolic content. All of these results were observed [17]. A score of 9 represented the pH function that was ideal for growth and yielded the maximum value for biomass production. The appropriate pH function for algae growth varies depending on a number of parameters, such as the varieties of algae being grown, the type of culture media being used, and the method being used to cultivate the algae in the laboratory. The suppression of photosynthetic activity at very high pH was shown to be the cause of the observed drop in growth, and this was due to the fact that there was no available carbon dioxide for the algae’s metabolism at these pH levels [17]. 

The capacity of *A. platensis* to adapt to high pH values is quite unique, as it allows the species to flourish in extremely alkaline conditions [49]. From 9 up to 10.5, it varies. Researchers have investigated how the levels of spirulina production fluctuate depending on the pH function in the environment (8, 9, 10, 11, and 12) [23,24]. After inoculating the first growth with 70 mL/700 mL of culture media and growing it at a temperature of 30 °C, with a light intensity of 26 μmoL photons/m^2^/s, the maximum dry weight (0.0853 g/20 mL) was attained. At a pH of 10, the high concentrations of proteins (425 mg/g) and carbohydrates (97 mg/g) can be explained by the fact that the activity of all the enzymes necessary for photosynthesis and respiration is maximized at this pH level. Spirulina thrives in alkaline environments with a pH ranging from 8.3 to 11, and the pH value has a direct impact on the rate at which oxygen is produced. The optimal range for photosynthesis is between 8 and 10 with a maximum pH of 11.5, marking the point at which oxygen generation slows to a crawl. The ideal pH range, which corresponds to the optimal value for photosynthesis, is between 8 and 10 [47]. 

### 4.8. Volume and Concentration of the Inocula

The preparation of inocula necessitates the continued culture of concentrated spirulina, and the strain being used should have a relatively high proportion of coiled filaments (less than 25% straight filaments) [1,45]. To cut down on the acclimation time and the less-than-ideal conditions that exist at the beginning of the planting process, an inocula that is pure, alive, and rapidly growing should be employed. It is essential to have an adequate inocula concentration in order to ensure a rapid acclimation phase and the continued viability of the culture. This is because of an inocula concentration that is too low results in the loss of algae as a result of photoinhibition [15]. In the study conducted by Ranjith and colleagues, the NRC-modified spirulina culture media were inoculated into a 250 mL Erlenmeyer flask. The flask included 100 mL of cultivation medium and inocula volume [50]. 

### 4.9. Agitation and Aeration

It is necessary to provide sufficient aeration and to move the culture in order to prevent the formation of thermal stratification, homogenize and maintain the suspension of the spirulina filaments, distribute nutrients evenly, and prevent the filaments from becoming clumped together. Only then can algae be cultivated successfully. The culture will benefit from the increased biomass of higher quality as a result of this. In addition to this, it assists in the uniform distribution of carbon dioxide and eliminates inhibitory chemicals such as oxygen. When ventilation is inadequate, there will be a reduction in both energy efficiency and the generation of biomass. As a result of the presence of air filled with holes, the floating cells that are found on the surface are subject to photoinhibition, which leads to a reduction in both growth and biomass production [1,47]. 

The agitation has the potential to have a detrimental impact on the growth of cultures; for example, little agitation might cause self-shading, while excessive agitation can cause culture stress [15]. Regular mixing, including the bubble mixing system, more than doubles the efficiency of photosynthesis by moving the spirulina cultures to homogenize the scattering of light and protect the filaments from photolysis that occurs during prolonged exposure to light. On the other hand, stirring at high speeds can damage the spirulina filaments and reduces the rate of photosynthesis [14,47,48,55]. Ahsan et al. [14] reported that the paddle wheel is the most common stirring device in commercial spirulina plants. Different devices may be used for mixing and stirring, such as pumps and pneumatic mixing systems, as well as gravity and manual agitation, and mechanical mixing can be accomplished by vibration, stirring, or oscillation [15,51]. 

### 4.10. Biomass Concentration

The cultivation of spirulina may be made more efficient by focusing on three criteria: production, quality, and cost. Spirulina biomass productivity may be enhanced by using the proper medium to lower the cost of spirulina production and improve the nutritional quality of biomass by boosting its iron content through the use of Fe-EDTA at a concentration of 10 mg/L. Spirulina production costs can be reduced by as much as 30 percent by utilizing a suitable medium [49]. The use of certain nutrients has an effect not only on production costs but also on the growth and the generation of biomass. The ability of algae to generate biomass across a given area in a given amount of time is referred to as their productivity [1]. Usharani et al. [53] evaluated the effects of a temperature at 30 °C and a pH of 9 on biomass production and total protein quantity in Spirulina isolated from an oily saline water environment in the Niger Delta. This environment produced the highest values of 4.9 mg/mL and 48.2 g/100 g, respectively. When compared to urea as a nitrogen source, the ammonium nitrate that was present in the medium provided an advantage to the metabolic efficiency of the algae. It was found that the growth rate and biomass values of *S. platensis* increased when the ammonium nitrate concentration increased. This was because there was an increase in the efficiency of the cultured cells to convert the reduced energy that resulted from photosynthesis into net growth. On the other hand, it gave the best biomass production at the lowest concentration of urea due to the inhibitory effect that urea has on the production of spirulina at concentrations of more than 0.3 g/L. Because of the rise in the urea content, rather than proteins, carbohydrates and lipids were accumulated in the body [17]. Spirulina is often grown in open ponds, which are advantageous in terms of cost, ease of construction and operation, and accessibility. The disadvantages of this method include a low biomass productivity of less than 15 g/m^2^/day, difficulty in maintaining optimal cultivation standards, high evaporation rates, and ease of contamination. Additionally, the growing medium has a significant impact on both biomass productivity and other important compounds [50]. Zarrouk’s medium offered the maximum biomass productivity (91.5 mg/L/day) for 13 days, whereas Hiri’s medium produced 80.5 mg/L/day and Jourdan’s medium produced 77.9 mg/L/day of spirulina biomass over the same time period. This comparison was carried out using three different culture media for spirulina [47]. It was reported that the development rate of *Spirulina* spp. is affected by salinity as well as the concentration of nutrients; specifically, the growth rate slows down when the salinity concentration increases. With sodium bicarbonate and sodium chloride salts, researchers were able to obtain the greatest amount of growth at the lowest possible salinity. It is well established that the biomass of algae may be doubled every three to four days through the straightforward process of cell division, which speeds up the pace of development. The presence of carbon dioxide in an aquaculture system contributes to an increase in the total cell concentration as well as the maintenance of a pH balance inside the facility [1,14]. The following sections will cover the many methods that are utilized to measure the concentration of biomass.

#### 4.10.1. Optical Density-Based Biomass Concentration Determination

There are two primary approaches that are utilized in the process of determining the concentration of biomass while the temperature, pH, and light intensities are held constant. The first technique involves determining the level of absorbance that algae have at a wavelength of 750 nm. The second process involves sifting and cleaning the biomass before drying it at 110 °C [49]. The growth of the spirulina cultivation was also evaluated by the optical density technique, with a wavelength of 750 nm as the measuring standard. Because of this, the result was indicative of the turbidity of the sample rather than the quantity of pigment that is there. The reason for this was because at this wavelength, the interference from chlorophyll pigment was at its lowest [15,52]. Madkour et al. [17] and Saeid and Chojnacka [54] measured the concentration of spirulina biomass in the cultivation media every three days by measuring the optical density at 560 nm. They also measured the concentration of the biomass of *Spirulina* spp. with a spectrophotometer. Samples were taken from the culture every day to determine the optical density at 560 nm [17,53]. In order to lessen the impact of dye adsorption, an optical absorbance measurement of algae culture was performed at 880 nm. On filters that had been pre-weighted and had a porosity of 0.7 μm, the dry weight of algae that had been dried was determined [47]. The turbidity was measured at 750 nm to provide an estimate of the number of spirulina cells per unit volume. The findings of Ranjith et al. [50] demonstrated that the LCMA medium was superior to Zarrouk’s standard medium (16.21%) in terms of the biomass productivity of *A. fusiformis*. Additionally, the LCMA medium had a higher yield for the dry extract, reaching 21.63% (Michael et al. 2018). According to Mogale [15], the biomass concentration was 0.08 g/L on day 0 of cultivation, and the maximum yield was 1.28 g/L on day 16 of the culture growth. Mogale [15] also indicated that the maximum yield occurred on day 16 of the culture growth.

#### 4.10.2. Dry Weight-Based Biomass Concentration Determination 

The growth rates of spirulina were measured in terms of the dry weight of biomass (mg/L), which was determined by filtering a sample of 0.45 µm. Between 27 and 33, there was an observed increase in growth rates as well as a higher concentration of biomass in a low-cost medium [17]. During the cultivation period of 40 days, the dry weight of spirulina *S. platensis* was measured and calculated every five days in order to determine the biomass. Five milliliters of each cultivating media were collected and filtered using sterile Whatman membrane filters with a pore size of 0.45 μm [53]. The optimum growth of spirulina was discovered at a salinity of 14 ppm, and the highest dry weight and growth rate of cells were 1.37 g/L and 0.25/day, respectively. On day 14, the culture cultivated at 30 °C had the highest growth and maximum cell concentration, which was 2.54 g/L [15]. 

### 4.11. Harvesting

Spirulina can be harvested in a variety of methods, but the early morning is the optimal time to do it since the biomass has a higher percentage of proteins at that point. Filtration, flotation, centrifugation, ultrasonic vibration, and other methods are utilized throughout the harvesting process. In addition, a filter or a mesh cloth with pores no smaller than 50 microns was utilized in order to collect the spirulina from the medium with an extremely high efficiency of around 95% [1,40]. After collecting the biomass through filtration, we first cleaned and washed it to dispose of excess salts, concentrated it to dispose of excess water, adjusted the acidity by adding an acid solution, spray-dried it, packaged it, and put it away somewhere dark to keep the pigments from deteriorating [14]. After 14 days of cultivation at 4 °C, Madkour et al. [17] harvested spirulina algal cells by centrifuging them at 10,000 rpm for ten minutes. In addition, Batista et al. [44] reported that the spirulina biomass was collected by filtration, centrifugation, and freeze-drying before being kept at a temperature of −20 °C until it was examined. According to Lemes et al. [46], the biomass of spirulina was sterilized at a temperature of 65 °C for 15 min and filtered using nylon filters first before being put through the process. 

## 5. Evaluation of the Potential for Microbial Contamination in Algae Cultivation Media

The contamination of spirulina cultures in open-air ponds by other algae is a serious problem; consequently, this topic has attracted the interest of a large number of researchers who are looking for appropriate conditions that selectively enhance the growth of algae required in pure cultures while preventing the growth of exotic organisms and zooplankton. It is possible to prevent contamination with chlorella algae by adding a high concentration of bicarbonate (0.2 M) and increasing the temperature of the cultivation ponds by covering them and heating them in the winter; it also prevents the growth of contaminated amoebae in the culture by adding ammonia at a concentration of 2 mM [14,24]. Protozoa such as amoeba and paramecium, which are common in spirulina cultures, are the most significant contributors to pollution at these facilities. Insects populate the culture in large numbers during the monsoon season, rendering the product unsuitable for human consumption. The culture is covered in plastic sheets to prevent pollution, and the pH level is maintained at a healthy range (between 9 and 11) to ensure that the circumstances on the culture are conducive to good health [1]. 

Salinity is the concentration of all dissolved salts in the cultivation media of *Spirulina* spp., which can tolerate different levels of salinity and high acidic function environments. This makes their environment unsuitable for bacteria, but some salt-loving bacteria such as haloalkaliphiles can grow including Halomonas, while salt has a negative effect on non-healing bacteria such as the genus Pseudomonas. Spirulina is beneficial to aerobic bacteria, such as Pseudomonas spp. and Micrococcus spp., because it can fix carbon dioxide in the biomass and release oxygen as a byproduct. These bacteria need oxygen to survive. Exogenous polysaccharides (EPSs) are produced by growing spirulina, which the bacteria use as a source of nutrition [15]. Because of this, the researchers emphasized the use of closed systems for cultivating spirulina, given that algal closed photobioreactors that protect against foreign particles in the air and the environmental systems that are contained within them, which results in 10 times the productivity and a reduction in pollution when compared to growing in the open air [1]. Chemical sterilization with sodium hypochlorite at 1% of the photobioreactor is performed in order to obtain a culture that is free of contamination with bacteria, viruses, and parasites. After 15 min, the photobioreactor is washed twice with tap water, and this is repeated [47,48,55]. Microbiological safety testing demonstrates that cultivating spirulina in a clean kitchen environment is not only possible but also safe for human consumption [23]. Because of their economic feasibility, open raceway pond systems are frequently employed in commercial micro-algae culturing. However, these systems are very susceptible to contamination by microorganisms. It was also found that the cultivation medium’s high acidity and alkalinity function inhibited the growth of most polluting organisms. This was due to the fact that the growth of spirulina causes an increase in the acidity function as a result of carbon dioxide absorption and the release of OH-into the cultivation medium [15]. 

## 6. Conclusions

*Spirulina* spp., which are a vivid blue-green color, are one of the earth’s earliest known forms of life. Its cells have a photovoltaic and prokaryotic cytoskeleton. One method for increasing the productivity of spirulina biomass is to use the appropriate media. It is also advised that diverse agricultural and industrial wastes are grown to reduce production costs and improve biomass nutritional quality. Critical discussion from the available published literature globally has also revealed that spirulina is able to respond to diverse pH levels and thrives in very alkaline environments. Furthermore, numerous ways for producing *Spirulina* spp. have been suggested, including open systems such as ponds and lakes, which are vulnerable to contamination by animals and otherworldly entities, and closed systems such as photovoltaic reactors, which are not. Because the bulk of the toxins contaminated by spirulina come from other species of hazardous algae that are combined with them when they are harvested, it is highly important to understand the processes used to produce spirulina at home. Furthermore, light, temperature, inoculation quantity, stirring speed, dissolved solids, pH, water quality, and the overall availability of micronutrients were identified as environmental parameters that influence spirulina productivity. Moreover, it was discovered that the culture medium’s extremely acidic and alkaline functions inhibited the growth of the majority of the contaminated organisms. As a consequence, we found and concluded from this article that it is feasible to develop *Spirulina* spp., which is economically significant in a variety of ready-made agricultural media and low-cost agricultural and industrial wastes. It is critical to fulfilling its growth and reproduction needs, which include a specified temperature, light intensity, acid function, movement, ventilation, and the acquisition of the highest potential biomass concentration. It is non-toxic and safe for human consumption, and its growth in high acidity functions prevents the growth of most organisms that are harmful to the environment, and these are the reasons why its cultivation is desired for the purpose of cultivation, which is to reap many nutritional and medicinal benefits. Despite the fact that the nutritional, environmental, and social advantages of spirulina have been collected from a variety of published literature, it is still reasonable to conclude that spirulina production is limited to a small number of natural areas. As a result, a rising number of scientists and experts all around the world are asking for more widespread spirulina production.

## Figures and Tables

**Table 1:** Classification of *Arthrospira platensis*, known as *Spirulina*.

Common Names	Taxonomic Classes
Bacteria	Domain
Eubacterla	Kingdom
Cyanobacteria	Phylum
Cyanophyceae	Class
Oscillatoriophycideae	Sub-class
Oscillatorlales	Order
Osellatorlaceae	Family
*Arthrospira*	Genus
*A. platensis*	Species

## Data Availability

Not applicable.
